# Acute Hepatitis E in the US Today Occurs in Diverse Patient Populations: Case Reports

**DOI:** 10.14740/gr602e

**Published:** 2014-05-02

**Authors:** Abimbola Aderinto-Adike, Mary R. Schwartz, Howard P. Monsour

**Affiliations:** a Houston Methodist Hospital, Texas, USA

**Keywords:** Acute hepatitis E, Developed countries, Organ transplant, Renal failure

## Abstract

Hepatitis E has long been thought of as an infection confined to the developing world. However, there has been an increased incidence of locally acquired cases in developed countries especially in transplant patients. Our first case is a 56-year-old Caucasian female post-heart transplant patient who presented with diarrhea and abdominal pain. She was found to be acutely infected with hepatitis E and progressed to stage 3 liver fibrosis. Our second patient was an otherwise healthy 76-year-old Vietnamese female who presented with abdominal pain, jaundice and fatigue. She was diagnosed with acute hepatitis E complicated by acute renal failure. There have only been a few reported cases of acute hepatitis E complicated by renal failure.

## Introduction

There is emerging data of locally acquired hepatitis E virus (HEV) infections in developed countries with the great majority of these patients being organ transplant patients [1]. These patients are often asymptomatic with the only clue being a modest rise in the transaminases into the 100 - 200 range [2]. In developing countries, HEV1 and HEV2 are the most common genotypes transmitted via a fecal or oral route, especially via contaminated water [3]. However, in developed countries, HEV3 and HEV4 genotypes are the most common locally acquired genotypes. HEV3 and HEV4 are transmitted via zoonosis and are felt to be transmitted to humans through consumption of uncooked animal products especially pigs, deer and boars [1, 3]. We present two cases of locally acquired acute hepatitis E seen at our institution. Both patients had exposure to deer and pigs.

## Case Reports

### Case 1

A 56-year-old female living in Louisiana presented with a two-month history of diarrhea and abdominal pain which worsened one week prior to presentation. She had undergone orthotopic heart transplantation in 1989 for post-partum cardiomyopathy during which time she was infected with hepatitis C. The patient received a second heart transplant in 2011 due to graft failure. She was on prednisone, tacrolimus and mycophenolate mofetil. She had no history of travel outside of the United States. Her family members engaged in deer hunting.

At the time she was seen in our clinic, she was tachycardic and hypotensive with a heart rate of 109 beats/min and a blood pressure of 89/56 mmHg. Laboratory studies at the time included a total bilirubin 9.4 mg/dL, alanine aminotransferase (ALT) 82 U/L and aspartate aminotransferase (AST) 60 U/L. Stool studies for *Clostridium difficile*, ova and parasites, *Campylobacter jejuni*, Cryptococcus, Shigella and Salmonella were negative. PCR RNA for Cytomegalovirus (CMV) and epstein-barr virus (EBV) were unremarkable. Hepatitis E IgM and IgG antibodies were positive at the time of presentation. HEV RNA by PCR assayed six weeks after presentation was undetectable.

**Figure 1:**
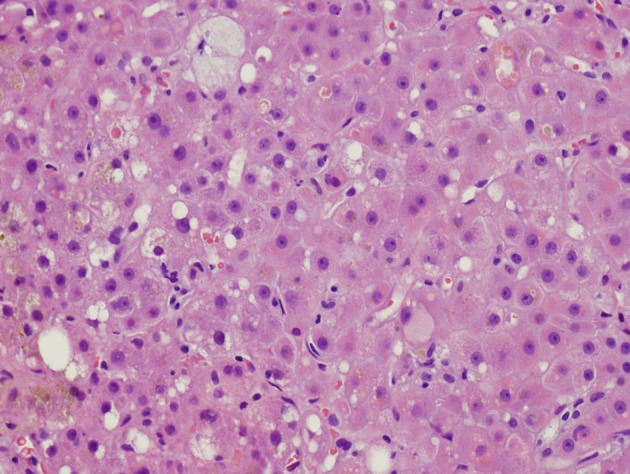
Initial liver biopsy showing lobular hepatitis, focal mild canalicular and intracellular cholestasis, focal intracellular fibrinogen, and mild steatosis (H and E, × 200).

Despite supportive management, the diarrhea persisted. Colonoscopy showed only diverticulosis. Random biopsies of endoscopically unremarkable mucosa showed no colitis. MRI of the abdomen showed no mass or biliary ductal dilation. Transjugular liver biopsy showed an active hepatitis with stage 1-2 fibrosis (Fig. 1). The hepatitic changes were felt to be related to hepatitis E given the absence of portal lymphoid aggregates typically seen in chronic hepatitis C.

**Figure 2:**
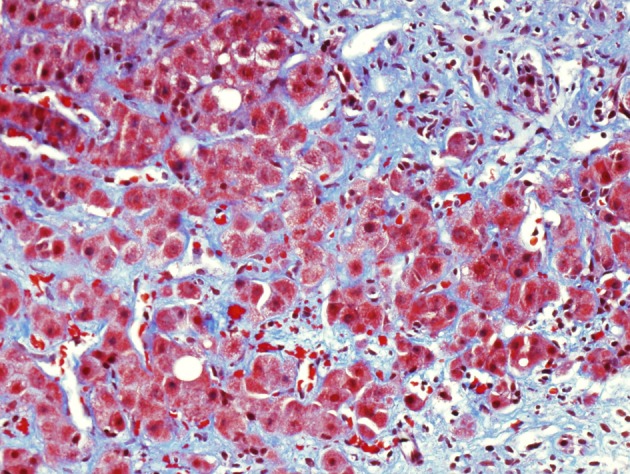
Follow-up liver biopsy, trichrome stain showing marked portal and perisinusoidal fibrosis.

Immunosuppressive therapy with tacrolimus was reduced from 2 mg to 1.5 mg daily. In addition, she was treated with oral ribavirin 600 mg twice daily for two months. At follow-up, the diarrhea and abdominal pain had resolved. Laboratory values included total bilirubin 1.9 mg/dL, AST 82 U/L and ALT 82 U/L. Follow-up liver biopsy obtained three months after the initial biopsy showed stage 3 fibrosis and marked perisinusoidal fibrosis (Fig. 2).

### Case 2

A 76-year-old Vietnamese female living in Texas presented with a three-week history of generalized weakness, subjective fevers, right upper quadrant abdominal pain, nausea, vomiting, loss of appetite and yellowing of her skin. The patient had moved to the United States 20 years earlier. Her last travel outside of the US was to Vietnam ten months prior to presentation. She had no significant past medical history. On dietary history, it was found that the patient ate pig liver and had been exposed to deer meat.

On physical examination, she was noted to have hepatomegaly without splenomegaly. Serum creatinine was 5.6 mg/dL, AST 122 U/L, ALT 204 U/L, alkaline phosphatase 1,159 U/L and total bilirubin 17 mg/dL. WBC was 11.48 × 10^3^/µL, hemoglobin 8.1 g/dL, MCV 80.9, platelets 163 × 10^3^/µL, INR 1.2 and albumin 2.8 g/dL. MRI of the abdomen and abdominal ultrasound with Doppler were unremarkable. HIV antibody was negative. Acute panel for hepatitis A, B, C and D was also negative. Hepatitis E IgM and IgG antibodies were positive. Hepatitis E PCR was assayed two weeks after admission and was undetectable. CMV IgM and IgG were positive, with serum CMV PCR assay 351 copies/mL. One week later, repeat CMV PCR was unremarkable. Autoimmune panel that included anti-smooth muscle and anti-mitochondrial antibodies, ANA, p-ANCA and c-ANCA were all unremarkable.

**Figure 3:**
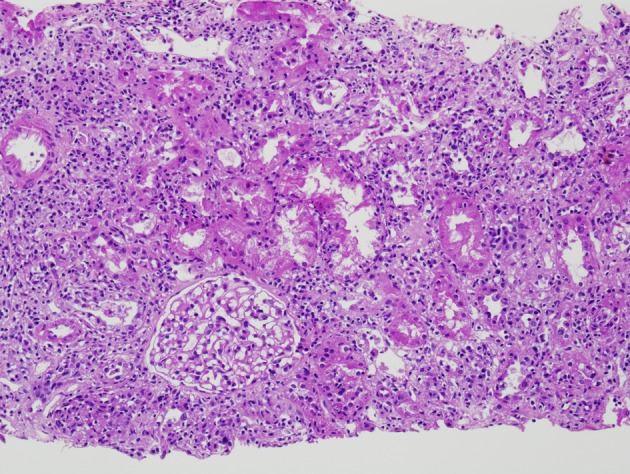
Initial renal biopsy showing a small discrete focus of interstitial lymphohistiocytic inflammation (H and E, × 100).

**Figure 4:**
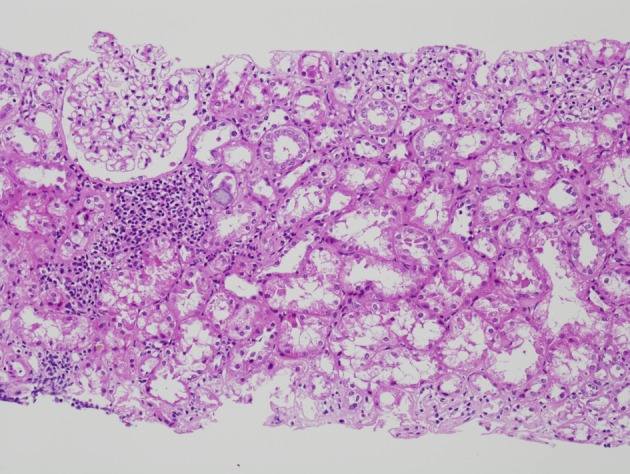
Repeat renal biopsy showing marked diffuse interstitial infiltration by lymphocytes (H and E, × 100). On immunochemical stain (not shown here), the lymphocytes were predominantly CD3 positive T cells with a predominance of CD8 positive T cells.

The initial renal biopsy showed a small focus of interstitial lymphohistiocytic infiltration (Fig. 3). A follow-up renal biopsy obtained seven weeks later (Fig. 4) showed evidence of severe interstitial lymphohistiocytic inflammation with extensive tubular injury and rare eosinophils. No immune deposits or vasculitis were seen. Direct immunofluorescence for IgG4 was negative.

Bone marrow biopsy showed no lymphoma, and flow cytometric immunophenotyping demonstrated no clonal cells or aberrant T cell antigen expression. Alkaline phosphatase isoenzyme assay demonstrated a liver fraction of 83% and bone fraction of 17%.

**Figure 5:**
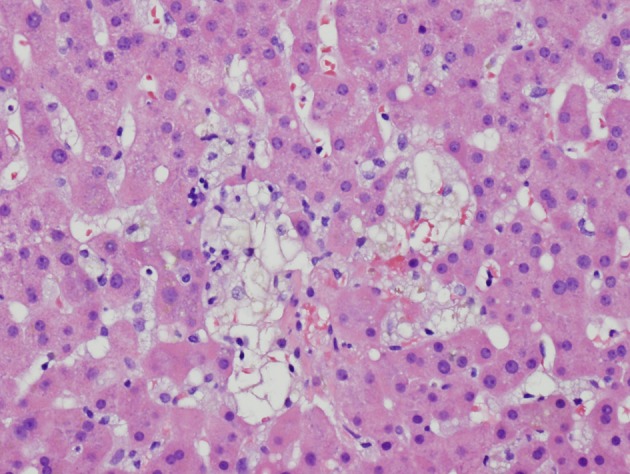
Liver biopsy showing focal hepatocyte dropout (H and E, × 200)

Transjugular liver biopsy showed very focal mild portal lymphocytic infiltration with rare eosinophils, a small focus of apoptotic hepatocytes, scattered tiny foci of individual hepatocyte dropout and focal minimal intracellular cholestasis (Fig. 5). No granuloma, CMV inclusion, significant steatosis, or fibrosis was seen. Immunohistochemical stain for CMV antigen was negative.

The patient was treated supportively for hepatitis E complicated by acute renal failure. She required intermittent blood transfusions and dialysis throughout her hospital admission. She eventually developed hospital-acquired pneumonia with acute respiratory insufficiency, ST-elevation myocardial infarction and cardiogenic shock, and died five weeks after her initial presentation.

## Discussion

Our first case is a heart transplant patient with acute hepatitis E. ALT and AST values had been normal pre- and post-transplant with values in the 20 - 30 range. About 20 months post-transplant, she presented with profuse diarrhea, worsening jaundice, pruritus, abdominal pain and elevated transaminases. The liver biopsy at the time of her presentation with acute hepatitis showed prominent cholestasis and hepatitic changes likely manifestations of acute hepatitis E. The absence of portal lymphoid aggregates, a characteristic finding in chronic hepatitis C, argues against worsening chronic hepatitis C. The stage 1-2 fibrosis in the liver biopsy at the time of presentation with acute hepatitis E was unchanged from the fibrosis stage in a previous biopsy obtained two years earlier. Follow-up liver biopsy obtained three months later showed significantly increased fibrosis, with stage 3 fibrosis and marked perisinusoidal fibrosis. It is possible that the patient now has chronic hepatitis E as well. About 60% of solid organ transplant patients infected with HEV fail to clear the virus, and eventually progress to chronic hepatitis [2]. Two important risk factors for the development of chronic hepatitis E appear to be use of tacrolimus (as compared to cyclosporine) and thrombocytopenia [2]. Our patient was on tacrolimus and had an additional risk factor of chronic liver disease from hepatitis C.

Our second case is a patient with acute cholestatic hepatitis E complicated by acute non-oliguric renal failure requiring intermittent dialysis. Development of renal failure in patients with cholestatic hepatitis, although presumably rare, markedly increases their morbidity [4, 5]. Our patient had a MELD-Na score on admission of 34. There have been reported cases of acute infectious hepatitis complicated by renal failure and severe hemolytic anemia in glucose-6-phosphate dehydrogenase (G6PD) deficient patients [5]. However, there have only been a few cases of acute hepatitis E infection associated with acute renal failure in this subset of patients with G6PD deficiency [4, 6]. In this group of patients, severe hemolysis results in excess production of hematin and bilirubin causing tubular obstruction [6, 7]. In our patient, renal failure was also complicated by mild CMV viremia. However, there was no evidence of CMV infection in either the kidney or liver biopsies. Hence, the etiology of renal failure in our patient was felt to be largely secondary to acute hepatitis E infection. The underlying pathophysiology for development of renal failure in patients with active hepatitis infection include: 1) acute tubular necrosis with a systemic inflammatory response state; 2) increased circulation of nephrotoxins (hematin, bilirubin and bile salts) in the presence of hepatic injury; 3) a hepatorenal-like state with decreased effective circulating volume in the presence of hyperbilirubinemia [4]. Our patient had many spherocytes on peripheral blood smear suggestive of a hemolytic anemia (LDH 1,301 U/L) and was thought to have an auto-immune hemolytic anemia. G6PD level was normal (15.9 U/g hemoglobin). However, normal G6PD level during active hemolysis does not exclude G6PD deficiency.

A diagnosis of acute hepatitis E is made by detecting HEV IgM antibodies in the serum. In immunocompromised patients, HEV antibodies may be falsely negative and HEV RNA should be tested in these patients [8]. Of note, HEV PCR was undetectable in both of our patients despite positive HEV IgM and IgG serologies. Our patients likely had acute on chronic infection given the timeline of onset of symptoms and their initial presentation with positive IgM and IgG HEV antibodies. The positive predictive value of HEV PCR declines in the days following an infection at which time the HEV IgM antibody is more likely to be positive and to be the main diagnostic indicator of infection [9]. In addition, there is significant variability in the accuracy of HEV PCR tests between laboratories [5, 9]. A case report by Yoo et al confirms the difficulty of establishing a diagnosis of a hepatitis E infection given the variability of serologic assays in the United States [10].

Most cases of acute hepatitis E are self-limited and resolve within 4 - 6 weeks [8]. Viral clearance with ribavirin alone can often be achieved when used up to three months in patients who do not respond to supportive management [5, 8]. Some reports have described using interferon in addition to ribarvirin as treatment for acute HEV [10, 11]. Although symptoms of diarrhea and abdominal pain improved on ribavirin treatment, it is unclear whether our first patient had a response to ribavirin as repeat liver biopsy showed worsening fibrosis. Our second patient was not considered for treatment with ribavirin because of anemia and acute kidney injury.

### Conclusion

The diagnosis of hepatitis E should be considered in patients in high resource countries with dietary risk factors for exposure to hepatitis E. Hepatitis E should also be considered in immunocompromised patients, especially organ transplant recipients, irrespective of age or travel history when patients present with acute diarrhea, hyperbilirubinemia and elevated aminotransferases.

## Abbreviations

HEV: hepatitis E virus
ALT: alanine aminotransferase
AST: aspartate aminotransferase
CMV: cytomegalovirus
EBV: epstein-barr virus
MELD: model for end stage liver disease
